# Child with acute lobar nephronia

**DOI:** 10.4103/0971-4065.70847

**Published:** 2010-07

**Authors:** M. Vijayakumar, N. Prahlad, G. Nandhini, N. Prasad, S. Muralinath

**Affiliations:** Department of Pediatric Nephrology, Mehta Children’s Hospital, Chennai - 600 031, India; 1 Department of Pediatric Surgery, Mehta Children’s Hospital, Chennai - 600 031, India; 2 Department of Pediatric Radiology, Mehta Children’s Hospital, Chennai - 600 031, India

**Keywords:** Interpretation, lobar nephronia, pyelonephritis

## Abstract

A five-year-old girl child presented to us with a history of two weeks high grade fever treated outside with intensive antibiotic therapy for an ultrasound abdomen finding of hypoechoic lesion in the midpole of the left kidney. As fever and sonographic findings persisted, a CT abdomen was done, which showed features of lobar nephronia but reported as Wilm’s tumor. Child underwent open biopsy and the diagnosis of lobar nephronia was confirmed. Child was continued on antibiotics and fever and sonographic findings improved.

## Introduction

Acute lobar nephronia (ALN) is a severe focal bacterial infection affecting one or more lobules of the kidney and is also termed acute focal bacterial nephritis. Rosenfield et al., first described it in 1979.[1,2] This condition is being increasingly diagnosed as a result of advancement of imaging technology. ALN presents as a localized nonliquefactive inflammatory renal bacterial infection which typically involves one or more lobes or lobules of the kidney. ALN as a renal infection is placed in between acute pyelonephritis (APN) and renal abscess in its natural history. The typical clinical presentation include fever, flank pain, leucocytosis, pyuria and bacteriuria which are again features of renal abscess and APN.[3] Ultrasonogram (USG) done by an expert sonologist and contrast enhanced computed tomography (CT) are useful to clinch the diagnosis. Technetium 99 m dimercaptosuccinic acid (DMSA) scintigraphy can show photon deficient areas on evaluation for APN which are later confirmed by CT studies.[4] But, DMSA studies are more useful in the evaluation of the child on follow-up than for primary diagnosis.[2] Problem comes when it can be misdiagnosed as space occupying lesion of the kidney wherein it becomes difficult to convince the parents of the diagnosis and hence may need renal biopsy and even open renal biopsy for confirmation, for future follow-up and legal issues.

We report a patient of ALN misreported as Wilm’s tumor, open renal biopsy confirming ALN and the child improved with continuation of antibiotic therapy.

## Case Report

A five-year-old female child presented to us with high grade fever of two weeks duration. Child was treated elsewhere with oral antibiotics since two weeks. Initially, the child had acute fever with evidence of infection as evidenced by polymorphonuclear leucocytosis. Child had received amoxycillin, gentamycin and cotrimoxazole in pediatric doses during the above said period. Urine culture done outside on the fourth day of antibiotics was sterile. Child also had more than 10 WBC/HPF in the urine and CRP was positive. As fever persisted for more than 10 days the general practitioner did USG of the abdomen, blood and repeat urine cultures. The cultures were sterile but USG abdomen showed a hypoechoic lesion in the midpole of the left kidney and was referred to us for further care.

**Figure 1:**
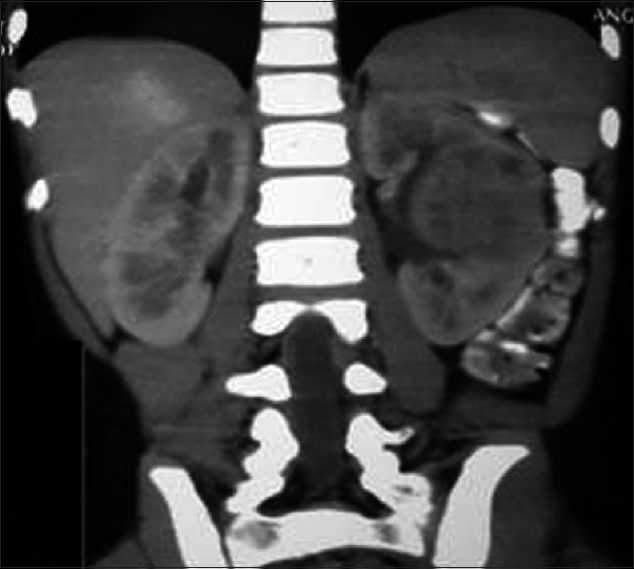
Contrast enhanced computed tomography picture of the lesion in the midpole of left kidney-along the column of bertini from papilla to cortex with calyceal effacement and poor enhancement of IV contrast

**Figure 2:**
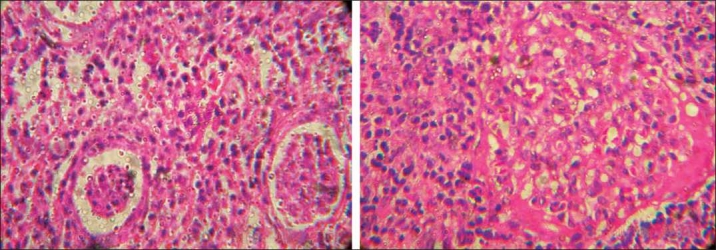
Light microscopy (H and E) of renal tissue showing dense interstitial and glomerular infiltrate of lymphocytes, plasma cells and neutrophils, sheets of histiocytes are seen focally. There is no evidence of malignancy

**Figure 3:**
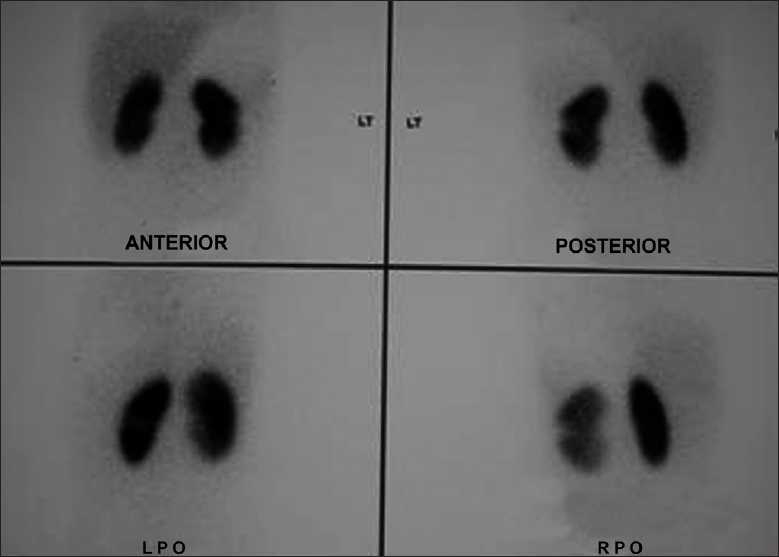
DMSA scan showing a wedge like defect in the middle of left kidney-healed inflammation with scar and the defect of the biopsied area

On admission, child had high grade fever, left loin pain, dysuria and severe vomiting. Child was toxic and hydration was poor. There was no evidence of shock and child had pallor. Vital signs were normal and BP was 92/70 mm/Hg in the right upper limb. No palpable mass per abdomen but left renal tenderness was present. Other systemic examination was essentially normal. Laboratory evaluation showed more than 10 WBC/HPF in urine, pus cells, pus cell casts and nonnephrotic proteinuria. Urine and blood cultures repeated were sterile. Polymorphonuclear leucocytosis, elevated ESR and positive CRP were noted. Fortunately, her renal functions were normal. USG abdomen showed hypoechoic lesion of 2 cm diameter near the hilum of the left kidney which was diagnosed as ALN by our radiologist. Child was treated with IV fluids, antiemetics and tolerable oral diet. She had IV antibiotics, ceftrioxone and tazobactam combination, amikacin and ofloxacin for seven days, as antibiotics could not be decided due to sterile cultures. Fever spikes were persisting and the repeat USG abdomen showed persistence of the lesion. Fearing a renal abscess and to decide on drainage of the pus from the site of abscess, a contrast enhanced CT abdomen was done outside our center. The CT scan showed a moderately well defined hyperdense lesion of 4 × 3cm in the interpolar region of the left kidney in the cortex and medulla with focal necrosis [Figure 1]. The surface appeared puckered. The lesion had moderate enhancement with focal irregular peripheral nonenhancing area. Further it was distorting the interpolar calyceal collecting system. Mild thickening of the left latero-conal and posterior para-renal fasciae was noted. Few enlarged lymph nodes were seen in the left renal hilum. There was double renal artery supply to the left kidney. The radiologist outside opined this as Wilm’s tumor with paraaortic lymphadenopathy for us to confirm with histopathology. The pediatric surgical team was involved in the management from this stage as a diagnosis of Wilm’s tumor was given by the outside radiologist, which was disputed by our radiologist. The CT findings had liquefactive areas which was suspected as pus in the centre of the lesion needing drainage as per our radiologist but the outside radiologist thought it as a necrotic area of a suspected Wilm’s tumor. Parents were counselled and open biopsy was carried out. The lesion measured 4 × 3 cm in the interpolar region of the left kidney in the cortex and medulla with distortion of the collecting system. The lesion showed a bluish area of inflammation, from which few drops of pus were drained, and a wedge biopsy was taken from the discoloured region. Histopathological examination of the renal tissue showed features of dense interstitial and glomerular infiltrates of lymphocytes, plasma cells and neutrophils. Sheets of histiocytes were seen focally. There was no evidence of malignancy and the impression was subacute inflammation with histiocytic reaction [Figure 2]. Child was continued on IV antibiotics for total two weeks and full dose oral antibiotics for one more week and later kept on chemoprophylaxis. Child became afebrile with the drainage of pus during the surgical procedure and on continuation of antibiotics by 10^th^day of admission. On follow-up, a DMSA scan was done to evaluate the progress in the child which showed left kidney had mildly reduced uptake with a small photopenic defect in the lateral border [Figure 3]. Right kidney had adequate uptake with smooth rounded cortical margins. After one month, a voiding cystourethrogram was done to rule out vesicoureteric reflux (VUR) and it was normal. Child is being followed up with chemoprophylaxis which is being planned for at least 6 months.

## Discussion

Acute lobar nephronia, otherwise termed as acute focal pyelonephritis or acute focal bacterial pyelonephritis is a nonliquefactive focal severe infection involving one or more of the renal lobules. It is a medical renal disease which presents as a febrile urinary tract infection. This falls in the spectrum of benign inflammatory lesions of the kidney with generalized pyelonephritis at one end, focal acute bacterial nephritis in the middle and renal abscess in the other end. All require intravenous higher antibiotics for a longer duration. As against the first and second in the spectrum, the last one the renal abscess requires a surgical drainage. About 25% of the children with ALN may progress into renal abscess if not treated with appropriate and adequate intravenous antibiotics.[5] Even the duration of the therapy differs with APN needing 14 days of intensive antibiotic therapy whereas ALN definitely needs three weeks of the same.[6]

The typical clinical presentations of ALN include fever, flank pain, leukocytosis, pyuria, and bacteriuria, which are similar to those with renal abscess or APN. The blood parameters are similar to that of any deep seated infections with neutrophilic leucocytosis and high C-reactive protein levels. The blood and urine cultures may or may not be sterile. The common organisms grown in a clean midstream sample or suprapubic aspirate are *E. coli, Klebsiella pneumoniae, Pseudomonas aeruginosa, Staphylococcal aureus*, etc.[6]

Imaging is vital in deciding on the further course of management of the disease. The basic ultrasonography is the gold standard in identifying the hypoechoic lesion in the kidney with or without areas of liquefaction. It has an observer variant but gives us adequate information by an expertise. It can mimic a spurious tumor. The nature of the lesion, consistency, liquefaction, perinephric extension, the inflamed thick renal capsule, perinephric space collection, involvement of hilum can be ascertained by USG abdomen. Hence, the use of ultrasound is of vital importance in the diagnosis and in follow-up of cases treated.[3,4]

Enhanced CT scan with IV contrast has got better anatomical delineation with more information on the following aspects, which goes in favour of ALN. The lesion presents as focal or global enlargement of the kidney. It primarily appears to radiate from the papilla to the cortex similar to columns of Bertini[7] which is not so in case of Wilms tumor which distorts the anatomy. The cortico-medullary differentiation is essentially blunted by edema with calyceal effacement. Due to edema, there is less vascularity of the lesion, and hence, gets poor enhancement by the IV contrast. The other features of inflammation include obliteration of renal sinus and perinephric fat planes as well as thickening of the pelvicalyceal wall and gerota’s fascia. This has to be delineated from the Wilm’s tumor where we get capsular enhancement by increased vascularity of the pseudocapsule formed by the compressed normal renal tissue around the tumor mass. There will be no features of inflammation, no delineation of clear planes but with involvement of the regional nodes.

Role of DMSA scan is important in follow-up of the cases as well as in identification of the lesion.[3,4] Here we get focal photopenic areas during the infective episode which may progress to form a renal scar with volume loss. This can be followed up subsequently as in any other case of pyelonephritis.

Once diagnosed to have ALN, child requires appropriate antibiotics by intravenous administration for a minimum period of two weeks followed by one week of oral antibiotics.[6] This condition is a slow responder to antibiotics and usually the fever settles only by the end of one week of IV antibiotics and it is necessary to have an image follow-up in the second week to ascertain that it doesn’t have pus collection in the centre of the lesion. A positive urine culture and *E. coli* growth is said to have poor response to the treatment.

Surgical intervention comes into play only in the 25% of the cases where the lesion turns to renal abscess.[5] This requires a surgical intervention to let out the pus. In case of a doubt of malignancy, then open biopsy of the lesion is warranted. The gold standard for diagnosis is to get a tissue diagnosis of the lesion. The other differential diagnosis for the ALN includes simple nephrogenic rests and xanthogranulomatous pyelonephritis (generalized disease).

## Conclusion

ALN is being recognized now more due to awareness of the condition and the excellent imaging services available. The availability of higher antibiotics, early identification, imaging support and appropriate treatment has started bringing down the incidence of scars in patients with acute lobar nephronia.
